# Supplementary material for: WordCommentsAnalyzer: A windows software tool for qualitative research
Source: F1000Res. 2018 Sep 4;7:536. Originally published 2018 May 3. [Version 2] doi: 10.12688/f1000research.14819.2 (PMC6137411; doi:10.12688/f1000research.14819.2)
Supplement: Supplementary file 3 [file f1000research-7-17614-s0002.tgz › b2a79496-4ada-47c4-9682-a99ce867836d.zip!/Eight Iranian Health Journals/Journal of Research in Rehabilitation Sciences _2.docx]

© Journal of Research in Rehabilitation Sciences . This work is licensed under CC BY-NC 4.0 (https://creativecommons.org/licenses/by-nc/4.0/).

Volume & Issue: دوره 13، شماره 3: 1396

Title: بررسی و مقایسه وضعیت بلندی اصوات با استفاده از پرسش‌نامه خودارزیاب Profile of Aided Loudness (PAL) در افراد جوان و سالمند کم‌شنوای متوسط و شدید استفاده کننده از سمعک

http://jrrs.mui.ac.ir/index.php/jrrs/article/view/2796

مقدمه: آزمون‌های ادیومتریک مرسوم، می‌توانند آسیب شنوایی یا عدم وجود آسیب را بررسی کنند، اما نمی‌توانند تأثیر ناتوانی یا معلولیت ناشی از کم‌شنوایی، میزان رضایت فرد از توان‌بخشی انجام شده یا تأثیر ناتوانی و یا معلولیت ناشی از نقص شنوایی را بررسی نمایند. پرسش‌نامه‌های خودارزیاب، این موارد را برای فرد کم‌شنوا مورد مطالعه قرار می‌دهند. پژوهش حاضر با هدف استفاده از پرسش‌نامه خودارزیاب Profile of Aided Loudness (PAL) جهت بررسی میزان رضایتمندی و بلندی اصوات برای افراد دارای سمعک و مقایسه کم‌شنوایی متوسط و شدید در افراد جوان و سالمند انجام شد. مواد و روش‌ها: در این مطالعه مقطعی، 20 نفر در محدوده سنی 40-20 سال به عنوان افراد جوان و 20 نفر در بازه سنی 80-60 سال به عنوان افراد سالمند شرکت نمودند. میزان رضایتمندي و بلندی از سمعک با استفاده از پرسش‌نامه خودازیاب PAL در گروه انتخابی مورد ارزیابی قرار گرفت. داده‌ها در نرم‌افراز تحلیل اطلاعات آماری تجزیه و تحلیل گردید. یافته‌ها: تفاوت معنی‌داری بین امتیازات بلندی در جوانان و سالمندان و مردان و زنان وجود نداشت. همچنین، اختلاف معنی‌داری در امتیازات بلندی صداهای آهسته مشاهده نشد، اما این تفاوت در صداهای متوسط و بلند معنی‌دار بود. تفاوت امتیازات رضایتمندی در کم‌شنوایی متوسط و شدید و در مردان و زنان معنی‌دار نبود، اما این امتیاز در جوانان و سالمندان اختلاف معنی‌داری را نشان داد. نتیجه‎گیری: مقیاس PAL جهت ارزیابی میزان بلندی درک شده توسط فرد در دو وضعیت بدون سمعک و با سمعک به کار می‌رود. نتایج به دست آمده از این پرسش‌نامه در مقایسه با مقادیر هنجار در تنظیم سمعک کاربرد دارد. همچنین، نتایج پژوهش حاضر نشان می‌دهد که احساس بلندی تحت تأثیر میزان کم‌شنوایی و احساس رضایتمندی تحت تأثیر سن قرار دارد.

Title: میانگین سرعت حرکات متناوب و متوالی هجایی در افراد 80-60 سال طبیعی شهر اصفهان

http://jrrs.mui.ac.ir/index.php/jrrs/article/view/2843

مقدمه: گفتار، مهارت حرکتی بسیار پیچیده‌ای است که به کنترل و هماهنگی سیستم‌های مختلف تنفس، آواسازی و تولید نیاز دارد. یکی از روش‌های اندازه‌گیری تکالیف حرکتی دهان که در هر واحد زمان محاسبه می‌شود، دیادوکوکینتیک می‌باشد. اطلاعات مربوط به ‌سرعت دیادوکوکینتیک، در تعیین وضعیت فیزیولوژیک تولید گفتار مفید است و شاخصی جهت ارزیابی، تشخیص و درمان بیماران دچار نقایص نورولوژیک سیستم گفتار محسوب می‌شود. سلامت عمومی می‌تواند با تغییر سن، بر سرعت و صحت حرکات بدنی و دهانی تأثیر بگذارد. با توجه به این که داده‌هایی از این معیار در سالمندان فارسی زبان در دسترس نیست، مطالعه حاضر با هدف تعیین سرعت حرکات متناوب (Alternating motion rate یا AMR) و سرعت حرکات متوالی (Sequential motion rate یا SMR) در سالمندان 60 تا 80 ساله طبیعی شهر اصفهان انجام گرفت. مواد و روش‌ها: 42 شرکت‌ کننده توالی‌های /pa/، /ta/ و /ka/ را به مدت 5 ثانیه و تناوب هجایی /pataka/ را به فاصله یک دقیقه استراحت، سریع و درست تکرار نمودند. نمونه‌ها با استفاده از ضبط کننده دیجیتال ضبط گردید و سپس سرعت بر حسب هجا بر ثانیه مورد محاسبه قرار گرفت. یافته‌ها: میانگین سرعت تکرار هجاهای /pa/، /ta/ و /ka/ به ترتیب 06/4، 90/3 و 66/3 هجا بر ثانیه بود. روند کاهش سرعت از جایگاه قدام به خلف مشاهده شد. میانگین AMR هجایی /pataka/ نیز 58/1 هجا بر ثانیه به دست آمد. نتیجه‎گیری: نتایج مطالعه حاضر در جهت جبران بخشی از کمبود اطلاعات طبیعی، نتایج توصیفی از سرعت دیادوکوکینتیک سالمندان فارسی زبان اصفهانی را ارایه نمود تا قضاوت بهتر و مطمئن‌تری از مقادیر غیر طبیعی بودن سرعت حرکات گفتاری سالمندان 60 تا 80 سال و اختلال حرکتی گفتار آن‌ها امکان‌پذیر شود. نتایج به دست آمده مؤید تفاوت‌های بین زبانی دیادوکوکینتیک، کندی سرعت در سالمندی و کاهش سرعت حرکت در خلف دهان می‌باشد.‎

Title: مقایسه فعالیت الکترومیوگرافیک عضلات منتخب کمربند شانه‌ای حین انجام ضربه Forehand topspin در بازیکنان تنیس روی میز حرفه‌ای با و بدون ابتلا به سندرم گیرافتادگی شانه

http://jrrs.mui.ac.ir/index.php/jrrs/article/view/2940

مقدمه: آسيب‌های شانه در ورزشكاران تنیس ‌روی ‌میز و ساير ورزش‌هايي که اندام فوقانی بالاتر از سطح شانه فعالیت می‌کند، بسيار شايع است. از طرف دیگر، عملكرد ضعيف يا نامناسب عضلات شانه، با بروز آسيب ارتباط دارد. هدف از انجام تحقیق حاضر، مقایسه سطح فعالیت عضلات منتخب کمربند شانه‌ای بازیکنان تنیس ‌‌روی ‌‌میز حرفه‌ای در دو گروه سالم و دارای سندرم گیرافتادگی شانه در حین اجرای ضربه Forehand topspin بود. مواد و روش‌ها: 30 بازیکن تنیس روی میز حرفه‌ای مرد 20 تا 28 ساله به ‌صورت هدفمند انتخاب شدند و در دو گروه با و بدون ابتلا به سندرم گیرافتادگی شانه قرار گرفتند (هر گروه 15 نفر). سپس فعالیت سطحی عضلات منتخب کمربند شانه‌ای در حین انجام ضربه Forehand topspin، از سمت غالب اندام فوقانی نمونه‌ها ثبت گردید. یافته‌ها: سطح فعالیت عضله سراتوس انتریور (007/0 = P) و سوپرااسپیناتوس (001/0 = P) در گروه مبتلا نسبت به گروه سالم کاهش معنی‌داری داشت. سطح فعالیت عضله تراپزیوس فوقانی نیز در گروه مبتلا نسبت به گروه سالم افزایش معنی‌داری را نشان داد (090/0 = P). در همین راستا، تفاوت معنی‌داری بین دو گروه در سطح فعالیت عضلات تراپزیوس تحتانی (301/0 = P)، دلتوئید قدامی (314/0 = P) و دوسر بازویی (291/0 = P) مشاهده نشد. نتیجه‌گیری: بيماران دارای علایم گيرافتادگي شانه، سطح فعاليت غیر طبیعی را در عضلات این ناحیه هنگام اجرای ضربه Forehand topspin نشان مي‌دهند. نتایج به دست آمده، اين تئوري را كه گيرافتادگي شانه ممكن است با تغيير در سطح فعاليت عضلات اسكاپولوتوراسيك و گلنوهومرال مرتبط باشد، تصديق مي‌كند.

Title: تأثیر یک دوره آموزش بدمینتون بر جبران فعالیت‌های بنیادی در کودکان مبتلا به عقب‌ ماندگی حرکتی

http://jrrs.mui.ac.ir/index.php/jrrs/article/view/2880

مقدمه: مهارت‌های حرکتی در کودکان با گذراندن مراحل رشد و نمو به بیشترین میزان ممکن برای پیشرفت در هر فرد نزدیک می‌گردد. با این وجود، رسیدن به این هدف به صورت خودکار حاصل نمی‌شود. از این‌رو، توجه به فعالیت‌های بنیادی در کودکان ضروری می‌باشد؛ چرا که عدم دسترسی به سطح پیشرفته این حرکات، فعالیت‌های دوران کودکی را کاهش می‌دهد و مانع دستیابی به حرکات تخصصی می‌گردد. هدف از انجام مطالعه حاضر، بررسی تأثیر مهارت‌های بدمینتون بر جبران عقب‌ماندگی فعالیت‌های بنیادی در کودکان بود. مواد و روش‌ها: شرکت‌کنندگان این پژوهش را دختران 7 تا 12 ساله شهر تهران که با عقب‌ماندگی حرکتی مواجه بودند، تشکیل داد. در ابتدا و انتهای تحقیق، پیش‌آزمون و پس آزمون با استفاده از آزمون حرکات درشت Ulrich (2-Test of Gross Motor Development یا 2-TGMD) به عمل آمد. گروه آزمایش به مدت 12 هفته تحت آموزش مهارت‌های بدمینتون قرار گرفتند. یافته‌ها: گروه آزمایش در مهارت‌های جابه‌جایی، به مراتب عملکرد بالاتری را نسبت به گروه شاهد که بدون تمرین در سطح فعالیت‌های روزمره نگهداشته شده بودند، نشان داد (001/0 = P). همچنین، یافته‌ها در رابطه با مهارت‌های دستکاری نیز تکرار گردید که بیانگر تأثیر تمرین بود (001/0 = P). نتیجه‌گیری: تأکید بر لزوم جبران عقب‌ماندگی حرکتی در سنین مدرسه ضروری می‌باشد و آموزش مهارت‌های بدمینتون، تأثیر مثبتی در جبران عقب‌ماندگی فعالیت‌های بنیادی کودکان دارد.

Title: بررسی اثربخشی برنامه Son-Rise بر بهبود تعاملات اجتماعی و ارتباط کودکان مبتلا به اختلال اوتیسم

http://jrrs.mui.ac.ir/index.php/jrrs/article/view/2891

مقدمه: اختلال طیف اوتیسم، توانایی فرد برای برقراری ارتباط با دیگران و پاسخ مناسب به محیط بیرون را تحت تأثیر قرار می‌دهد. از جمله رویکردهای درماني در کودکان مبتلا به این اختلال، برنامه Son-Rise است. مطالعه حاضر با هدف بررسی اثربخشی برنامه Son-Rise بر بهبود تعاملات اجتماعی و ارتباط کودکان مبتلا به اختلال اوتیسم انجام شد. مواد و روش‌ها: این پژوهش يك کارآزمایی شبه بالینی با طرح پیش‌آزمون و پس‌آزمون همراه با گروه شاهد بود. 30 کودک مبتلا به اختلال اوتیسم با استفاده از روش نمونه‌گیری در دسترس انتخاب شدند. كودكان به صورت تصادفی در دو گروه مداخله برنامه فشرده Son-Rise و شاهد (تحت مداخلات توان‌بخشی رایج) قرار گرفتند (در هر گروه 15 كودك). از ویرایش دوم آزمون Gilliam Autism Rating Scale (2-GARS) و مقیاس رشد اجتماعی Vineland به عنوان پیش‌آزمون و از آزمون 2-GARS برای ارزیابی مجدد استفاده گردید. داده‌ها با استفاده از آزمون‌های Independent t، Mann-Whitney، Paired t و Wilcoxon مورد تجزيه و تحليل قرار گرفت. یافته‌ها: برنامه Son-Rise در مقایسه با مداخلات توان‌بخشی رایج، سبب بهبود تعامل اجتماعی کودکان مبتلا به اوتیسم شد (001/0 = P). اگرچه تفاوت معنی‌داری در مقایسه نتایج ارتباط شرکت‌کنندگان در دو گروه مشاهده نگردید (110/0 = P). نتیجه‎گیری: می‌توان نتیجه‌گیری کرد که برنامه Son-Rise باعث بهبود تعامل اجتماعی کودکان مبتلا به اوتیسم می‌شود، اما تأثیر این برنامه بر بهبود تعامل اجتماعی این گروه از کودکان، به بررسی بیشتر و تحقیقات مداخله‌ای تجربی آزمایشی با تعداد بیشتری از کودکان مبتلا به اوتیسم نیاز دارد.

Title: بررسی تأثیر یک دوره برنامه تمرینی ترکیبی بر عوامل آمادگی جسمانی دانش‌آموزان ناشنوای پسر: مطالعه کارآزمایی بالینی تصادفی مقدماتی

http://jrrs.mui.ac.ir/index.php/jrrs/article/view/2914

مقدمه: ناشنوایان رفتارهاي حركتي و اجتماعي متفاوتي دارند كه البته بعضي از آن‌ها مشهود است. اين مشخصه‌ها بيشتر در حفظ تعادل بدن، هماهنگي، قدرت و استقامت مشاهده می‌شود که در واقع، می‌توان این مشخصه‌های متفاوت را در حیطه شاخص‌های آمادگی جسمانی طبقه‌بندی کرد که ناشنوایان در مقایسه با همتایان عادی خود در این شاخص‌ها از آمادگی کمتری برخوردار هستند. بنابراین، مطالعه حاضر به بررسی تأثیر یک دوره برنامه تمرینی ترکیبی (استقامتی و ثبات مرکزی) بر عوامل آمادگی جسمانی دانش‌آموزان ناشنوا پرداخت. مواد و روش‌ها: جامعه آماری پژوهش شامل 24 دانش‌آموز ناشنوای پسر بود که به ‌صورت تصادفی در دو گروه تجربی (12 نفر) و شاهد (12 نفر) قرار گرفتند. تعادل ایستا با استفاده از آزمون ارزیابی خطاهای تعادل، تعادل پویا با استفاده از آزمون تعادلی Y، استقامت عضلانی تنه با استفاده از آزمون Sorensen، استقامت عضلانی شکم با استفاده از آزمون دراز و نشست و استقامت قلبی- تنفسی با استفاده از آزمون پله اندازه‌گیری شد. گروه تجربی یک دوره برنامه تمرینی ترکیبی را به مدت 8 هفته و هر هفته سه جلسه (هر جلسه 60 دقیقه) انجام داد. به ‌منظور تحلیل داده‌ها، از آزمون‌های Independent t و Paired t استفاده شد. یافته‌ها: بر اساس نتایج آزمون Paired t، در گروه تجربی اختلاف معنی‌داری بین نتایج پیش‌آزمون و پس‌آزمون تعادل ایستا، پویا، استقامت عضلانی تنه، استقامت عضلانی شکم و استقامت قلبی- تنفسی مشاهده شد (001/0 > P). نتایج آزمون Independent t در زمینه تأثیر تمرینات ترکیبی (استقامتی و ثبات مرکزی) بر تعادل ایستا، پویا، استقامت عضلانی تنه، استقامت عضلانی شکم و استقامت قلبی- تنفسی پسران، تفاوت معنی‌داری را بین گروه‌های تجربی و شاهد نشان داد (001/0 > P). نتیجه‌گیری: با توجه به نتایج پژوهش حاضر می‌توان گفت که مجموعه‌ای از تمرینات استقامتی و ثبات مرکزی، منجر به بهبود وضعیت جسمانی در دانش‌آموزان ناشنوا می‌شود.

Title: مطالعه‌ای بر ویژگی‌های پیکری منتخب دختران و پسران در دوران کودکی و نوجوانی

http://jrrs.mui.ac.ir/index.php/jrrs/article/view/2965

مقدمه: ویژگی‌های ساختاری بدن، اطلاعات مهمی همچون رشد طبیعی بدن، نوع فعالیت‌ بدنی متناسب با اندازه بدن، استعدادیابی ورزشی و همچنین، پیش‌بینی ابتلا به برخی بیماری‌ها مانند مشکلات قلبی- تنفسی و سرطان را در اختیار پزشکان، مراقبان سلامت، مربیان و والدین قرار می‌دهد. پژوهش حاضر با هدف تعیین و مقایسه میانگین ویژگی‌های قامتی شامل طول قد در دو موقعیت خوابیده و ایستاده، قد نشسته و نیز نسبت قد نشسته به قامت ایستاده انجام شد. مواد و روش‌ها: از میان کل 315987 دانش‌آموز ابتدایی تا دبیرستان شهر اصفهان، 768 کودک و نوجوان به‌ روش خوشه‌ای انتخاب شدند و بر حسب گروه سنی و جنسیت، در چهار گروه قرار گرفتند. متغیرهای قامتی با متر استاندارد ثبت شد. داده‌ها با استفاده از آزمون‌های Levene و One-way ANOVA و همچنین، آزمون‌های تعقیبی مورد تجزیه و تحلیل قرار گرفت. یافته‌ها: قد خوابیده، روند مشابهی با قامت ایستاده داشت و از قامت ایستاده بیشتر بود. با وجودی ‌که اندازه قد نشسته پسران بیشتر از دختران گزارش گردید، اما نسبت قد نشسته به قامت ایستاده پسران، نسبت به دختران کمتر بود. دختران و پسران نوجوان به ترتیب بیشترین و کمترین نسبت قد نشسته به قامت ایستاده را داشتند. تفاوت معنی‌داری بین نسبت قد نشسته به قامت ایستاده در گروه‌ها مشاهده گردید (001/0 > P). نتیجه‌گیری: تفاوت معنی‌داری بین میانگین قد خوابیده، قامت ایستاده و قد نشسته کودکان دختر و پسر وجود ندارد. از نظر میانگین قد نشسته به قامت ایستاده، نوجوانان دختر و کودکان، در یک طبقه و نوجوانان پسر در طبقه‌ دیگر قرار گرفتند. یافته‌ها با استانداردهای قامت مراکز کنترل و پیش‌گیری بیماری‌ها (Centers for Disease Control and Prevention یا CDC) مطابقت دارد.

Title: تأثیر دوازده هفته تمرینات عملکردی بر زاویه سر به جلو و شاخص‌های اسپیرومتری

http://jrrs.mui.ac.ir/index.php/jrrs/article/view/2858

مقدمه: سر به جلو، یکی از شایع‌ترین ناهنجاری‌های وضعیتی است که منجر به عدم سازماندهی عضلات مشترک بین گردن و شانه می‌شود و در نهایت، ظرفیت ششی و تنفس صحیح افراد را با مشکل مواجه می‌سازد. هدف از انجام پژوهش حاضر، بررسی تأثیر 12 هفته تمرینات عملکردی بر ناهنجاری سر به جلو و شاخص‌های اسپیرومتری در افراد مبتلا به این عارضه بود. مواد و روش‌ها: در این مطالعه نیمه تجربی، 40 فرد دارای ناهنجاری سر به جلوی بیشتر از 46 درجه به صورت هدفدار انتخاب شدند و به صورت تصادفی در دو گروه 20 نفره تجربي و شاهد قرار گرفتند. قبل و پس از 12 هفته تمرینات عملکردی (کششی- ثباتی و خوداصلاحی فعال- تکلیف محور)، زاویه سر به جلو با استفاده از روش عكس‌برداري از نماي نيم‌رخ و محاسبه زاویه خط میان تراگوس و 7C با خط عمودی (زاویه سر به جلو) اندازه‌گیری و شاخص‌های اسپیرومتری ثبت گردید. از آزمون تحلیل کواریانس جهت تجزیه و تحلیل داده‌ها استفاده شد (05/0 > α). یافته‌ها: در گروه تجربی، میانگین زاویه سر به جلو بعد از تمرینات به طور معنی‌داری کاهش یافت و از 14/4 ± 25/55 به 24/4 ± 30/51 درجه رسید (001/0 > P). همچنین، افزایش معنی‌داری در ظرفیت‌های تنفسی Forced expiratory volume (FEV1) (از 50/4 ± 50/87 به 51/4 ± 95/92 درصد)، Forced vital capacity (FVC) (از 04/6 ± 05/89 به 83/4 ± 10/94 درصد) و Peak expiratory flow (PEF) (از 08/4 ± 55/86 به 00/4 ± 65/91 لیتر بر دقیقه) مشاهده گردید (001/0 > P)، اما چنین تغییراتی در گروه شاهد ایجاد نشد. نتیجه‌گیری: انجام تمرینات عملکردی به عنوان یک روش ساده و غیر تهاجمی جهت اصلاح عارضه سر به جلو و در نتیجه، بهبود وضعیت تنفسی در افراد دارای این ناهنجاری توصیه می‌گردد.
